# LINC01158 works as an oncogene in glioma via sponging miR-6734-3p to boost CENPK expression

**DOI:** 10.1186/s12935-021-01931-x

**Published:** 2021-05-27

**Authors:** Zhenxing Sun, Naili Wei, Shenglian Yao, Guihuai Wang, Yaxing Sun, Zhenze Wang, Dan Yuan

**Affiliations:** 1 Department of Neurosurgery, School of Clinical Medicine, Beijing Tsinghua Changgung Hospital, Tsinghua University, Beijing, 102218 China; 2 Department of Neurosurgery, The First Affiliated Hospital of Shantou University Medical College, Shantou, 515041 Guangdong China; 3 School of Material Science and Engineering, University of Science and Technology Beijing, Beijing, China; 4 Department of Psychiatry, Zaozhuang Mental Health Center, Zaozhuang, 277103 Shandong China; 5 Department of Neurosurgery, Haicheng Zhenggu Hospital, Anshan City, 114200 Liaoning China; 6 Department of Nephrology, Beijing Luhe Hospital, Capital Medical University, No. 82, Xinhuanan Road, Tongzhou District, Beijing, 102218 China

**Keywords:** LINC01158, CENPK, MiR-6734-3p, Glioma

## Abstract

**Background:**

Long non-coding RNAs (lncRNAs) have been certified to play vital biological functions in glioma and have received considerable attention in the recent literature. Nonetheless, the role of LINC01158 in glioma remains to be elucidated.

**Methods:**

qRT-PCR, western blot and GEPIA database were applied for reporting the expression of CENPK and LINC01158 in glioma and the correlation between LINC01158 and CENPK expression. EdU, colony formation, CCK-8, caspase-3 activity and TUNEL assays probed the impacts of LINC01158 on glioma cell growth. Subcellular fractionation and FISH assays revealed the cellular distribution of LINC01158. Luciferase reporter and RIP assays examined ceRNA network of LINC01158, CENPK and miR-6734-3p.

**Results:**

LINC01158 and CENPK were both overexpressed in glioma and a positive regulation of LINC01158 on CENPK was corroborated. LINC01158 served a pro-proliferative and anti-apoptotic part in glioma by sponging miR-6734-3p to augment CENPK.

**Conclusion:**

LINC01158 enhances CENPK by serving as sponge for miR-6734-3p to facilitate glioma development, proposing LINC01158 as a new player in glioma.

**Supplementary Information:**

The online version contains supplementary material available at 10.1186/s12935-021-01931-x.

## Background

The incidence of malignancy attacking central nervous system is increasing year by year. In China, brain tumor is one of the top ten most common cancers [1, 2]. As one major part of brain tumors, glioma represents the most familiar primary fatal tumor among adults [3, 4]. Currently, chemoradiotherapy has been gradually worked as a substitute for surgical resection which once dominated the management of glioma, but the efficacy has not met the patients’ need. Despite of the methods of optimizing therapeutic regimen of glioma, the 2-year survival rate of glioma patients remains dreadful due to the elusive understanding of glioma pathophysiological mechanisms. Hence, it is needful to dissect the mechanism of glioma pathogenesis.

As elucidated from the data of human genomic sequence, only 2% transcripts have potent capacities of protein encoding, and the rest are noncoding RNAs (ncRNAs). NcRNAs can be primarily divided into two subtypes, including long noncoding RNAs (lncRNAs) and microRNAs (miRNAs), both of which exert multi-biological functions in the development of tumors [5, 6]. Depending on the circumstances, lncRNAs or miRNAs could be either oncogenic or tumor-suppressive during the onset and progression of cancers [7, 8]. For instance, lncNRA TTTY15, promotes the development of prostate cancer through sponging let-7 [9]. In gastric cancer, miR-133a-3p represses cell growth and metastasis through blocking autophagy-mediated glutaminolysis [10]. In glioma, actively participated lncRNAs have also been extensively documented. LncRNA ATB promotes malignancy in glioma by negatively regulating miR-200a [11]. LncRNA NEAT1, regulated by the EGFR pathway, contributes to glioblastoma progression through WNT pathway by scaffolding EZH2 [12]. Increased lncRNA H19 promotes the invasion, angiogenesis, and stemness of glioblastoma cells [13].

Mechanically, lncRNAs competed with certain downstream messenger RNAs (mRNAs) for miRNAs through acting as miRNA sponges to work in tumor progression [14]. For instance, LINC01296/miR-26a/GALNT3 constitutes ceRNA network to facilitate colorectal cancer progression through PI3K/AKT pathway [15]. LINC00511 counteracts the inhibition of miR-185-3p on E2F1 expression to speed up breast cancer tumourigenesis and stemness [16]. LncRNA DANCR serves as a ceRNA of miR-149 to mediate malignant progression of bladder cancer through augmenting MSI2 level [17]. In glioma, lncRNA CCDC26 [18], PVT1 [19], SNHG15 [20] and et al. have been considered as the contributors of glioma initiation and carcinogenicity. However, most lncRNAs have not been mechanically and functionally explained in glioma, which deserves more deep-going discussion.

In this study, we discovered that CENPK and LINC01158 were both increased in glioma cells and tissues, and positively correlated with each other. Functional assays indicated LINC01158 and CENPK was pro-proliferative to glioma cells. Mechanically, LINC01158 predominated in the cytoplasm was responsible for the increase of CENPK via sponge miR-6734-3p. LINC01158 facilitated glioma cell malignant phenotypes through elevating CENPK via its sponging on miR-6734-3p, which is of value for therapeutic strategies in glioma.

## Methods

### Cell culture

KeyGEN Biotech Company (Nanjing, China) was the supplier of human astrocytes (NHA), while four glioma cell lines (U87, SHG44, T98G and U251) were attained from American Type Culture Collection (ATCC). All cells were cultivated in RPMI-1640 medium (Invitrogen, Carlsbad, CA) with 10% fetal bovine serum (Gibco, CA) and at 37℃ in an humidified atmosphere with 5% CO_2_.

### Cell transfection

Three shRNAs devised for targeting LINC01158 (sh-LINC01158#1, sh-LINC01158#2, sh-LINC01158#3) and the non-targeting control (sh-NC) were synthesized and supplied by Vigene Biosciences (Shandong, China). The pcDNA3.1/CENPK vector for overexpressing CENPK was purchased from Sangon Biotech (Beijing, China). GenePharma (Shanghai, China) provided the mimics and inhibitor of miR-6734-3p or miR-4775 and negative controls (NC-mimics, NC-inhibitor). Cells cultured in 6-well plates underwent transfection with these plasmids as indicated using Lipofectamine 2000 (Invitrogen; Thermo Fisher Scientific, Inc.) referring to the supplier's instructions. 48 h later, the collected cells were prepared for further experiments.

### Quantitative real-time polymerase chain reaction (qRT-PCR)

Eastep®Super Total RNA Extraction kit (Promega, Madison, WI, USA) was applied for isolating total RNA from related cells. cDNA synthesis applying the Transcriptor First Strand cDNA Synthesis kit (Roche, Basel, Switzerland) was completed. qRT-qPCR employing GoTap®qPCRMaster Mix (Promega) was carried out under CFX96™ Real-Time PCR Detection Systems. 2^−ΔΔCt^ method was used for the estimation of the relative expression levels of object genes. 3 times tested specimen was required. The primer sequences were listed: LINC01158 F-primer, 5′-AATCACTGCAATTGAAGGAAAAA-3′ and R-primer, 5′-CCTTGTTTTCCAACCCTTAGACT-3′; CENPK F-primer, 5′-AATGTTGCCTACGTGACCCG-3′ and R-primer, 5′-TCCCCACCGTCACAAAAACA-3′; GAPDH F-primer, 5′-GGGAGCCAAAAGGGTCAT-3′, and R-primer, 5′-GAGTCCTTCCACGATACCAA-3’.

### Western blot

Protein isolation with lysis buffer was first executed, followed by electrophoretically separation via SDS-PAGE and then transferring onto PVDF membranes (Millipore, Billerica, MA). Subsequently, the membranes were blocked in 5% non-fat milk (Becton–Dickinson and Company, Suzhou, China) and bred overnight with primary antibodies at 4℃. Then, the membranes were co-incubated for 1 h with secondary antibody at room temperature. ECL chemiluminescent detection system (Thermo Fisher Scientific, Rochester, NY) were thereafter utilized for protein band visualization.

### Cell counting kit-8 (CCK-8) assay

U87 and T98G cells were plated at 3 × 10^3^ cells each well in a 96-well plate and overnight maintained. At 0, 24, 48, 72 and 96 h following cultivation, 10 μl of CCK8 solution was supplemented and the plates went on incubating at 37℃ for 4 h. The optical density (450 nm) was evaluated with a microplate reader (Synergy2, BioTek).

### Ethynyl deoxyuridine (EdU) incorporation assay

To assess U87 and T98G cell viability, EdU incorporation assays were implemented in strict line with the suggestions of an EdU kit (Roche). Zeiss AxioPhot Fluorescence Microscope (Carl Zeiss, Oberkochen, Germany) was utilized for capturing the images, followed by the quantification of EdU-positive cells via Image-Pro plus 6.0 software.

### Colony formation assay

Colony formation assay was conducted in U87 and T98G cells to assess their colony formation abilities. Following a 24 h-transfection, U87 and T98G cells were planted in 6-well plates with 2 × 10^3^ cells distributed per well. 12–14 days later, phosphate‑buffered saline (PBS) was applied for washing the cells twice, followed by cell fixation with paraformaldehyde and cell staining with 0.5% crystal violet solution for 20 min at room temperature. At length, the number of colonies (≥ 50 cells) was counted manually.

### TdT-mediated dUTP nick end-labeling (TUNEL) assay

The apoptosis of indicated U87 and T98G cells was analyzed by TUNEL assays under the Dead EndTM Fluorometric TUNEL System (Promega, Madison, WI, USA) in accordance with the manufacturer’s specification. Zeiss photomicroscope (Carl Zeiss, Oberkochen, Germany) was exploited to capture images, and TUNEL-positive cells selected from at least five random fields were counted and quantified.

### Caspase-3 activity assay

U87 and T98G cells underwent the determination of caspase-3 activity as instructed by a caspase-3 activity assay kit (Beyotime, Haimen, Jiangsu, China). In brief, cells were harvested, lysed with lysis buffer and incubated at 4℃. Following the centrifugation of the cell lysates for 15 min (12,000 g/min), the supernatants were attained, followed by the addition of Ac-DEVD-pNA (10 μl, 2 mM) and cultivation at 37℃ for 2 h. Finally, a microplate reader (WoYuan Tech, Hongkou, Shanghai, China) was employed for monitoring the absorbance at 405 nm.

### Cell cytoplasm/nucleus analysis

The expression of LINC01158 in the cytoplasm or nucleus of glioma cells was analyzed with the application of nuclear and cytoplasmic RNA PARISTM Kit (Ambion, Austin, TX). Glioma cells were gathered and re-suspended in the cell fraction buffer. Subsequently, qRT-PCR was employed for analyzing RNAs in the isolated supernatant and nuclear pellet.

### *Fluorescence *in situ* hybridization (FISH)*

FISH assay was utilized for detecting the distribution of LINC01158 in glioma cells. Genechem Co. Ltd. (China) supplied the fluorescent LINC01158 FISH probe. Using 4% paraformaldehyde, U87 and T98G cells were immoblized on slides and then co-incubated overnight with probes (50 nmol/L) at 37℃. DAPI was applied for counterstaining the nuclei of U87 and T98G cells. Fluorescent microscope (Olympus) was used for the acquirement of digital fluorescent photographs.

### Luciferase reporter assay

Beforehand, either wild type LINC01158 or matched mutant LINC01158 binding sites was inserted into pmirGLO luciferase reporter plasmids and the constructed plasmids were named as LINC01158-WT or LINC01158 MUT. Besides, the wild-type 3′-UTR of CENPK or mutant one was also cloned into the pmirGLO luciferase reporter vectors to construct CENPK-WT or CENPK-MUT reporter. Above recombinant reporters were co-transfected with miR-6734-3p mimics or NC-mimics into U87 and T98G cells. After 48 h transfection, Renilla (internal control) and firefly luciferase activities were subjected to the detection via Dual-Luciferase Reporter Assay System (Promega).

### Statistical analysis

Statistical analyses were achieved by the usage of SPSS 20.0 software (SPSS, Chicago, IL) and the data were expressed as mean ± SD. Student's *t* test or one-way/two-way ANOVA was employed for the significance determination of differences between groups, and the significance of statistics was assigned at P < 0.05 (*), P < 0.01 (**) or P < 0.001 (***).

## Results

### LINC01158 positively regulated CENPK and both were overexpressed in glioma

**Fig. 1:**
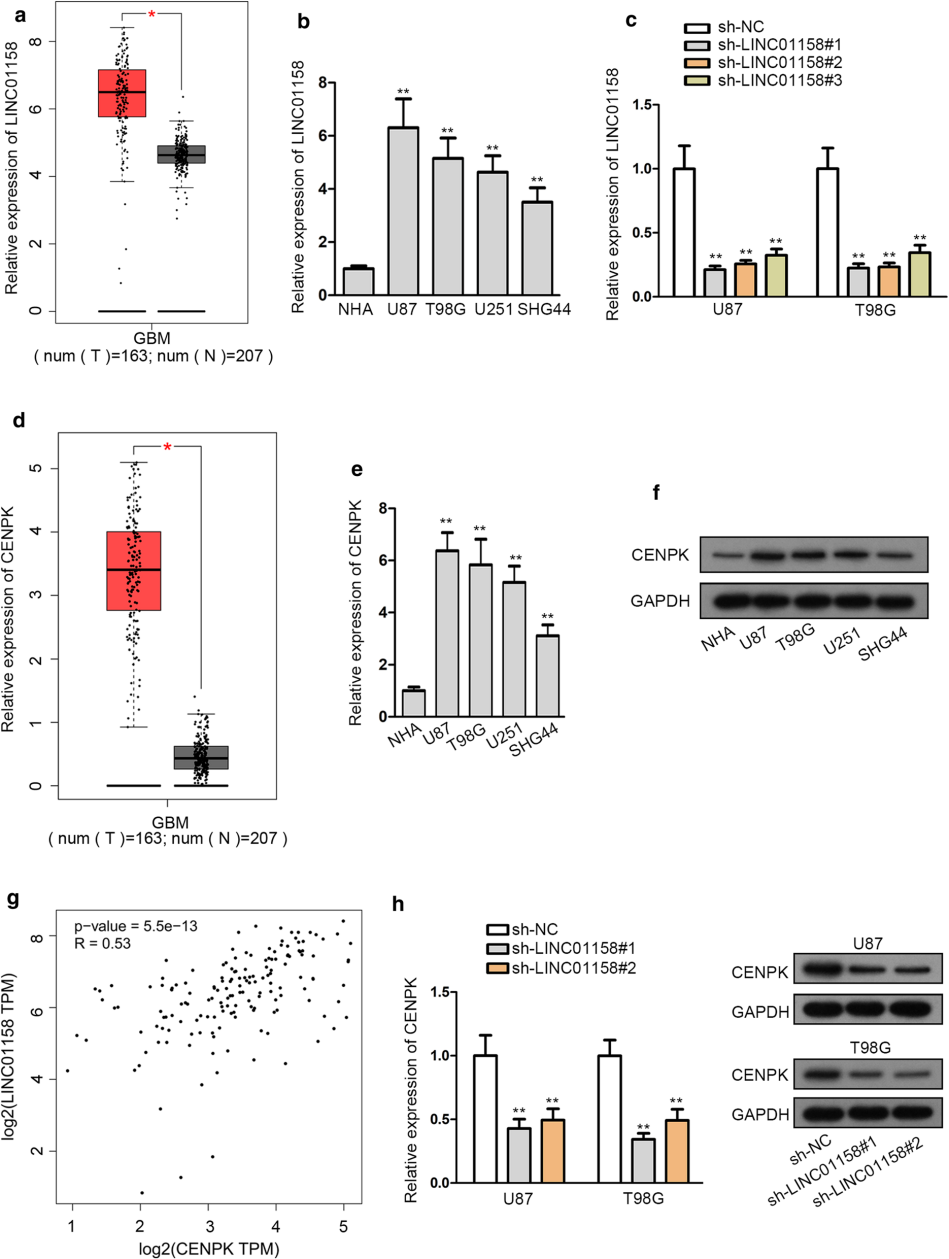
LINC01158 positively regulated CENPK and both were overexpressed in glioma. **a** Box plot of LINC01158 expression in GBM tissues and normal controls from GEPIA. **b** qRT-PCR measurement of LINC01158 in glioma cell lines and control NHA cells. **c** qRT-PCR detection of LINC01158 level of in glioma cells following LINC01158 interference. **d** Box plot of CENPK expression in GBM tissues and normal samples from GEPIA. (E) CENPK mRNA expression in glioma cells was under the assessment of qRT-PCR. **f** CENPK protein level in glioma cells was monitored applying western blot. **g** A positive correlation was found between LINC01158 and CENPK expressions from GEPIA. (H) CENPK mRNA and protein levels in glioma cells after silencing LINC01158 were examined by qRT-PCR and western blot. * and ** note P < 0.05 and P < 0.01, respectively

The enrichment of LINC01158 was probed and a dramatic increase of LINC01158 in GBM (glioblastoma multiforme) tissues was illustrated by the data from GEPIA database (Fig. 1a). Significantly, high LINC01158 level indicated an unfavorable prognosis in patients with glioma (including both GBM and low grade glioma) (Additional file 1: Fig. S1A). Meanwhile, LINC01158 was also proved to be upregulated in glioma cell lines compared to the normal NHA cells (Fig. 1b). Thereafter, we silenced LINC01158 expression in U87 and T98G cells by specific shRNAs for subsequent use (Fig. 1c). To figure out the downstream genes of LINC001158, we searched for the similar genes of LINC01158 in GBM tissues by using GEPIA. As a result, among the top ten genes correlated with LINC01158 in GBM, four (including RHEB, SUB1, RAD54B and CENPK) were protein-coding genes. Further, only CENPK could be apparently suppressed in both U87 and T98G cells in response to LINC01158 deficiency (Additional file 1: Fig. S1B). Besides, CENPK was found as an upregulated gene in GBM tissues via GEPIA data (Fig. 1d). Moreover, we also confirmed the elevation of CENPK at both mRNA and protein levels in glioma cell lines relative to NHA (Fig. 1e, f). Then, a positive correlation between LINC01158 and CENPK expression in glioma was plotted from GEPIA database (Fig. 1g). Further, we discovered that CENPK mRNA and protein levels were suppressed with LINC01158 silencing (Fig. 1h). Collectively, overexpressed LINC01158 positively regulated CENPK in glioma.

### Knockdown of LINC01158 suppressed glioma cell growth

**Fig. 2:**
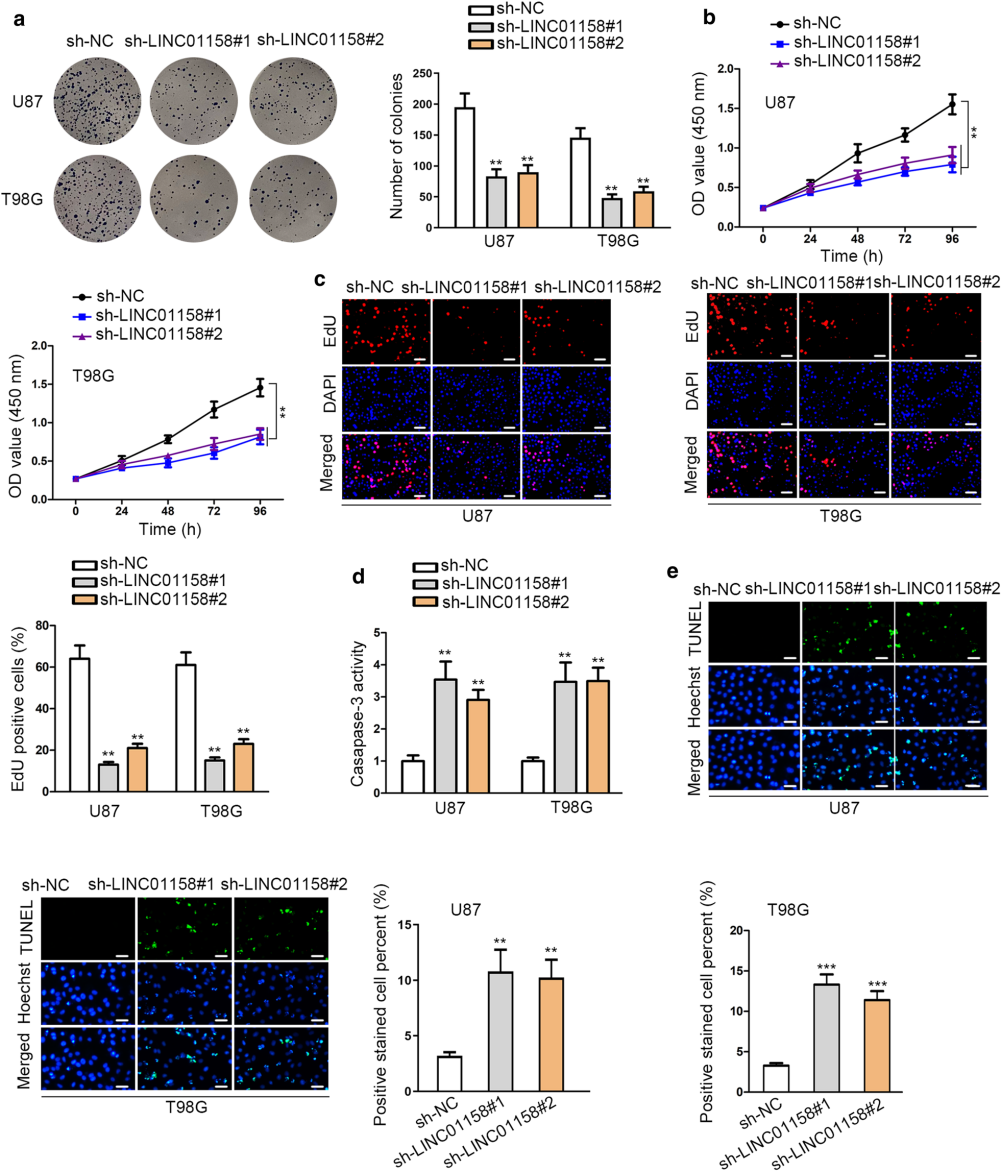
Knockdown of LINC01158 suppressed glioma cell growth. **a** Colony formation assay detected the proliferative potential of glioma cells following LINC01158 depletion. **b** CCK-8 assay was implemented for examining the viability of glioma cells with LINC01158 depletion or not. **c** EdU assay was used for checking the proliferation of glioma cells with LINC01158 depletion or not. Scale bar = 100 μm. **d**, **e** Caspase-3 activity and TUNEL assays (scale bar = 100 μm) determined the effect of LINC01158 depletion on glioma cell apoptosis. **and *** note P < 0.01 and P < 0.001, respectively

Then, we wanted to know the impact of LINC01158 on glioma development. As expected, glioma cells with depleted LINC01158 presented suppressed colony formation abilities (Fig. 2a). The results of CCK-8 assay illuminated the restrained cell viability in response to LINC01158 depletion (Fig. 2b). Likewise, EdU assay results elucidated that glioma cell proliferation was blocked upon LINC01158 inhibition (Fig. 2c). Caspase-3 activity assay showed that the absence of LINC01158 accelerated glioma cell apoptosis (Fig. 2d). Similar results could also be found by TUNEL assay (Fig. 2e). Taken together, LINC01158 was oncogenic for glioma development.

### LINC01158 functioned as a sponge for miR-6734-3p to enhance CENPK expression

**Fig. 3:**
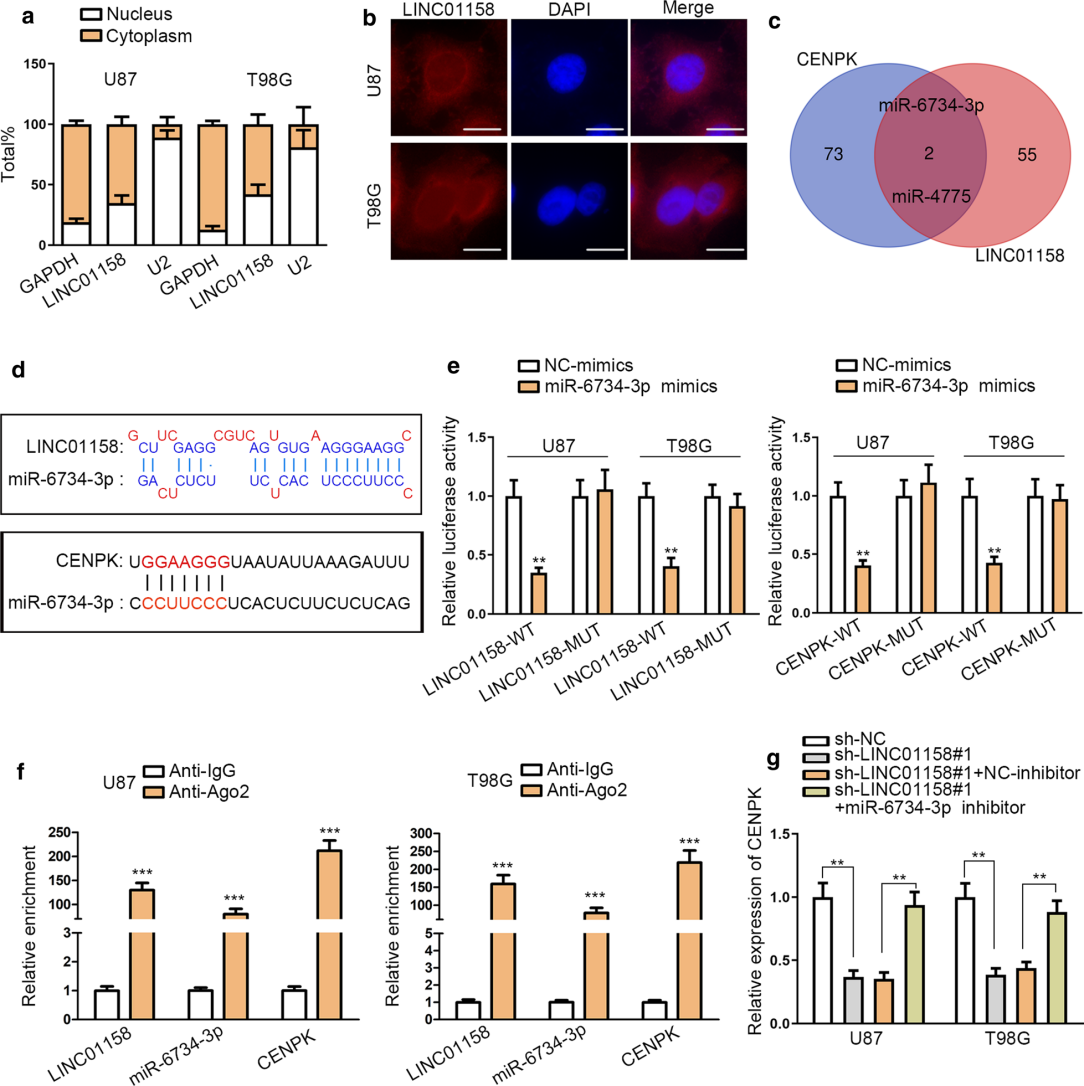
LINC01158 functioned as the sponge of miR-6734-3p to enhance CENPK expression. **a** Subcellular fractionation followed by qRT-PCR examined LINC01158 distribution in the nucleus or cytoplasm of glioma cells. **b** FISH assay determined the location of LINC01158 in glioma cells. Scale bar = 10 μm. **c** DIANA and miRDB predicted miR-6734-3p and miR-4475 as the potential partners of both LINC01158 and CENPK. **d** The miR-6734-3p binding sites within LINC01158 and CENPK were separately predicted by DIANA-lncBase and miRDB. **e** Luciferase reporter assay investigated the luciferase activity in glioma cells with miR-6734-3p mimics (or NC-mimics) and LINC01158-WT/MUT or CENPK-WT/MUT reporters. **f** RIP assay suggested LINC01158, miR-6734-3p and CENPK could associate with Ago2 protein. **g** qRT-PCR illustrated that downregulating miR-6734-3p reversed the suppressed trend of CENPK mRNA altered by LINC01158 depletion. **and *** note P < 0.01 and P < 0.001, respectively

To dissect LINC01158-mediated mechanism in glioma, the subcellular presence of LINC01158 was determined. Results from Subcellular fractionation and FISH assays indicated higher level of LINC01158 in the cytoplasm of U87 and T98G cells (Fig. 3a, b), indicating that LINC01158 might work as miRNA sponges in glioma. Then, we aimed to validate whether LINC01158 served as a ceRNA of CENPK via sponging certain miRNA. Hence, miRNAs which could interact with both LINC01158 and CENPK were searched with the assistance of DIANA-lncBase and miRDB. Resultantly, miR-6734-3p and miR-4775 were screened out (Fig. 3c). In addition, we unveiled that LINC01158 blockade resulted in the increase of miR-6734-3p level not that of miR-4775 in these two glioma cells (Additional file 1: Fig. S1C). Further, decreased CENPK level was also observed in both cells upon miR-6734-3p mimics not miR-4775 mimics (Additional file 1: Fig. S1D). Based on these data, miR-6734-3p was selected as the topic subsequently. MiR-6734-3p binding sites in LINC01158 and CENPK were shown in Fig. 3d. Luciferase reporter assay results indicated that upon the overexpression of miR-6734-3p, LINC01158-WT and CENPK-WT luciferase activities were reduced while no impacts were shown in the activities of LINC01158-MUT and CENPK-MUT reporters (Fig. 3e). Additionally, strongly enriched LINC01158, miR-6734-3p and CENPK were confirmed in Ago2 group relative to IgG group (Fig. 3f). Moreover, qRT-PCR analyzed that decreased CENPK expression due to LINC01158 silencing was restored when miR-6734-3p was further inhibited in glioma cells (Fig. 3g). Overall, LINC01158 sponged miR-6734-3p to augment CENPK expression in glioma.

### LINC01158 expedited glioma cell growth via * miR-6734-3p/CENPK * signaling

**Fig. 4:**
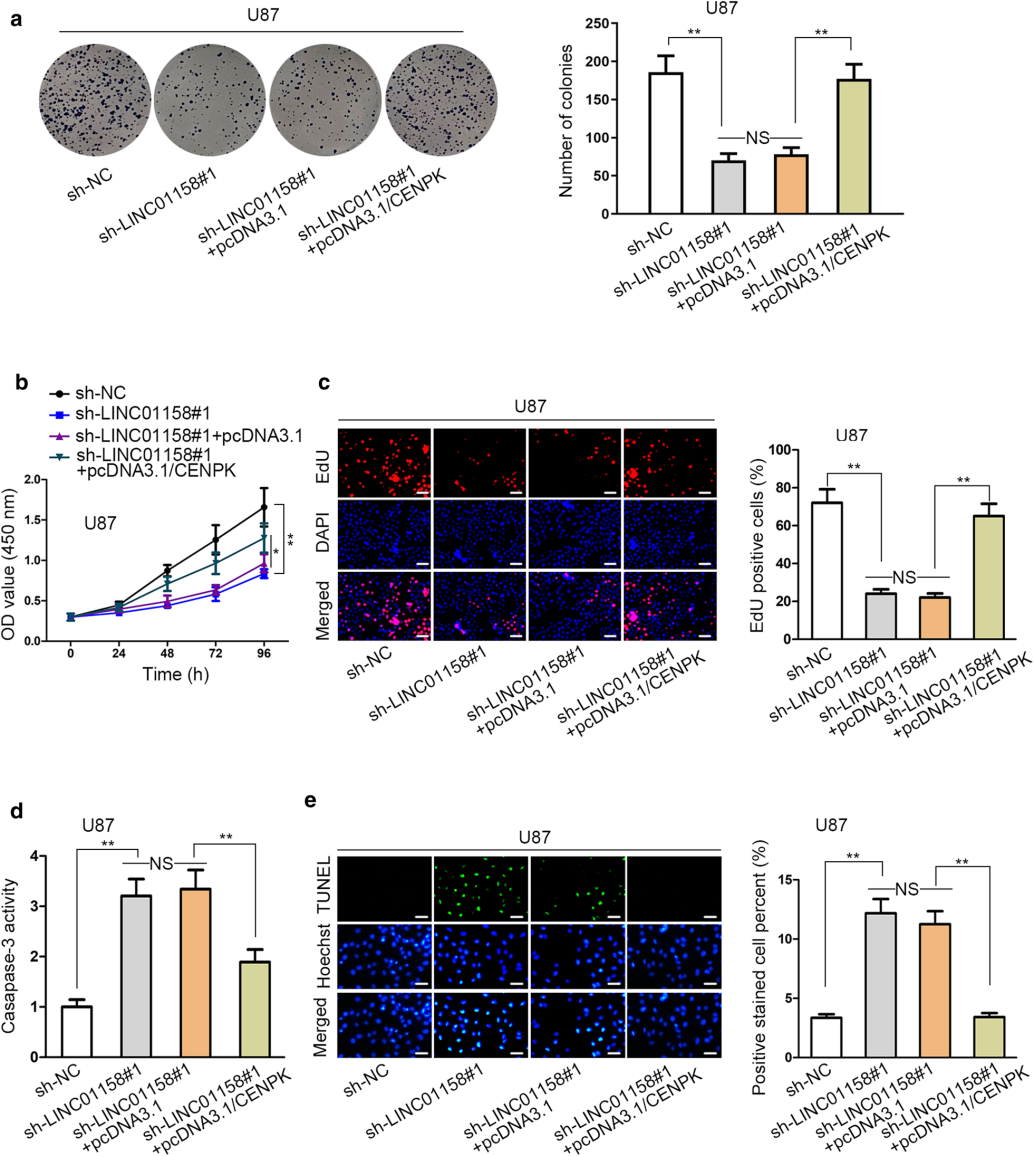
CENPK elevation rescued glioma cell proliferation and apoptosis altered by LINC01158 downregulation. **a** The proliferation of glioma cells under various conditions was monitored by colony formation assay. **b** Cell viability under different contexts was measured applying CCK-8 assay. **c** EdU analysis of glioma cell proliferation under different conditions. Scale bar = 100 μm. **d** Caspase-3 activity assay reflected glioma cell apoptosis following several indicted treatments. **e** TUNEL analysis of glioma cell apoptosis in response to diverse transfections. Scale bar = 100 μm. * notes P < 0.05, ** notes P < 0.01, and NS notes not significant

To further substantiate that LINC01158 aggravated malignancy in glioma through miR-6734-3p/CENPK signaling, we conducted a series of rescue assays. Overexpression efficiency of CENPK was ensured in pcDNA3.1/CENPK-transfected glioma cells by qRT-PCR (Additional file 2: Fig. S2A). Besides, it was disclosed that overexpressing CENPK strengthened colony formation ability and cell viability, and enhanced proportion of EdU-positive cells in glioma (Additional file 2: Fig. S2B–D), certifying CENPK as a growth-facilitator in glioma. Thereafter, we revealed that inhibiting miR-6734-3p counteracted the proliferation-restraining and apoptosis-accelerating effects of LINC01158 knockdown on glioma cells (Additional file 2: Fig. S2E-I). Then, we tested whether CENPK was required in LINC01158-regulated glioma development. Results manifested that the suppressed colony formation ability by administration of sh-LINC01158#1 was reverted using pcDNA3.1/CENPK (Fig. 4a). The declined cell viability upon LINC01158 depletion was recovered with CENPK augmentation (Fig. 4b). The retarded cell proliferation due to LINC01158 silencing was restored in response to the elevation of CENPK (Fig. 4c). With respect to glioma cell apoptosis, the outcomes of caspase-3 activity and TUNEL assays unveiled that cell apoptosis was enhanced because of LINC01158 repression, while the elevation of CENPK effectively attenuated the promoted trend (Fig. 4d, e). These data demonstrated that LINC01158 played a carcinogenic part in glioma depending on sequestering miR-6734-3p from CENPK.

## Discussion

All over the world, glioma has turned into a huge health burden for patients with this disease. Meanwhile, high morbidity of glioma is still dreaded. Therefore, it is of tremendous significance to unveil effective prognostic biomarkers and therapeutic targets to ameliorate the clinical outcomes of glioma patients. In recent years, lncRNAs have been evidenced to modulate gene expression at several processing levels to affect cancer development [23]. POU3F3 adjacent non-coding transcript 1(LINC01158; linc-POU3F3; PANTR1) was proved to facilitate colorectal cancer [24], gastric cancer [25] and hepatocellular carcinoma [26]. In present work, we unveiled LINC01158 was upregulated in glioma and its silencing hindered glioma cell growth.

Then, our study also identified Centromere protein K (CENPK) as the potential downstream of LINC01158 in glioma. Besides, we discovered CENPK expression was elevated in glioma, and the positive regulation of LINC01158 on CENPK expression was confirmed in glioma. It was previously documented that CENPK aggravated the development of hepatocellular carcinoma [21] and ovarian cancer [22]. In glioma, CENPK was linked to TCGA subtypes and tumor grades [27]. In this study, CENPK was confirmed to be increased in glioma cells and gain-of-function assays demonstrated CENPK as a growth-accelerator in glioma.

Further, we found that LINC01158 was mainly a cytoplasmic lncRNA in glioma. Since cytoplasmic lncRNAs often function as ceRNAs [28], here we recognized miR-6734-3p as the shared miRNA between LINC01158 and CENPK. More importantly, we testified that LINC01158 augmented CENPK expression in glioma cells through sponging miR-6734-3p. In current literatures regarding malignancies, miR-6734-3p was only reported to promote leukemogenesis by targeting p27 [29]. Herein, we proved that inhibiting miR-6734-3p could reversed the suppressive impact of silenced LINC01158 on glioma cell growth, implying it played as a tumor-inhibitor in gliomagenesis. In the end, the rescue assays validated that LINC01158 contributed to gliomagenesis depending on miR-6734-3p/CENPK pathway.

## Conclusion

Collectively, our work supported the oncogenic role of LINC01158 in glioma via miR-6734-3p/CENPK signaling. The current study indicated a novel role of LINC01158 as a potent target for glioma therapy.

## Supplementary Information

**Additional file 1: Figure S1.** (A) GEPIA data of the survival curve of patients with glioma (including both GBM and low grade glioma) possessing low or high LINC01158 level. (B) Impact of LINC01158 inhibition on the expression of RHEB, SUB1, RAD54B or CENPK in U87 and T98G cells was assessed by qRT-PCR. (C) The effects of LINC01158 depletion on miR-6734-3p and miR-4775 levels were under qRT-PCR examination. (D) The influence of miR-6734-3p and miR-4775 on the expression of CENPK was under the analysis of qRT-PCR. ** notes P < 0.01.

**Additional file 2: Figure S2.** (A) The expression of CENPK in glioma cells transfected with pcDNA3.1 or pcDNA3.1/CENPK was tested by qRT-PCR. (B-D) The impact of CENPK overexpression on the function of glioma cells was evaluated by colony formation, CCK-8 and EdU assays. (E-I) The rescuing effect of miR-6734-3p inhibition on the function of LINC01158-depleted glioma cells was estimated through CCK-8, colony formation, EdU, caspase-3 activity and TUNEL assays. ** notes P < 0.01.

## Data Availability

Related data and materials have been shown in the present manuscript and supplementary files.

## Abbreviations

lncRNAs: Long non-coding RNAs
mRNAs: Messenger RNAs
miRNAs: MicroRNAs
ceRNA: Competing endogenous RNA
ATCC: American Type Culture Collection
qRT-PCR: Quantitative real-time polymerase chain reaction
CCK-8: Cell counting kit-8
EdU: 5-Ethynyl-2′-deoxyuridine
TUNEL: TdT-mediated dUTP nick end-labeling
FISH: Fluorescence in situ hybridization
RIP: RNA immunoprecipitation
WT: Wild-type
MUT: Mutant
SD: Standard deviation
ANOVA: Analysis of Variance
