# Supplementary figures and images for: LINC01158 works as an oncogene in glioma via sponging miR-6734-3p to boost CENPK expression
Source: Cancer Cell Int. 2021 May 27;21:280. doi: 10.1186/s12935-021-01931-x (PMC8161569; doi:10.1186/s12935-021-01931-x)

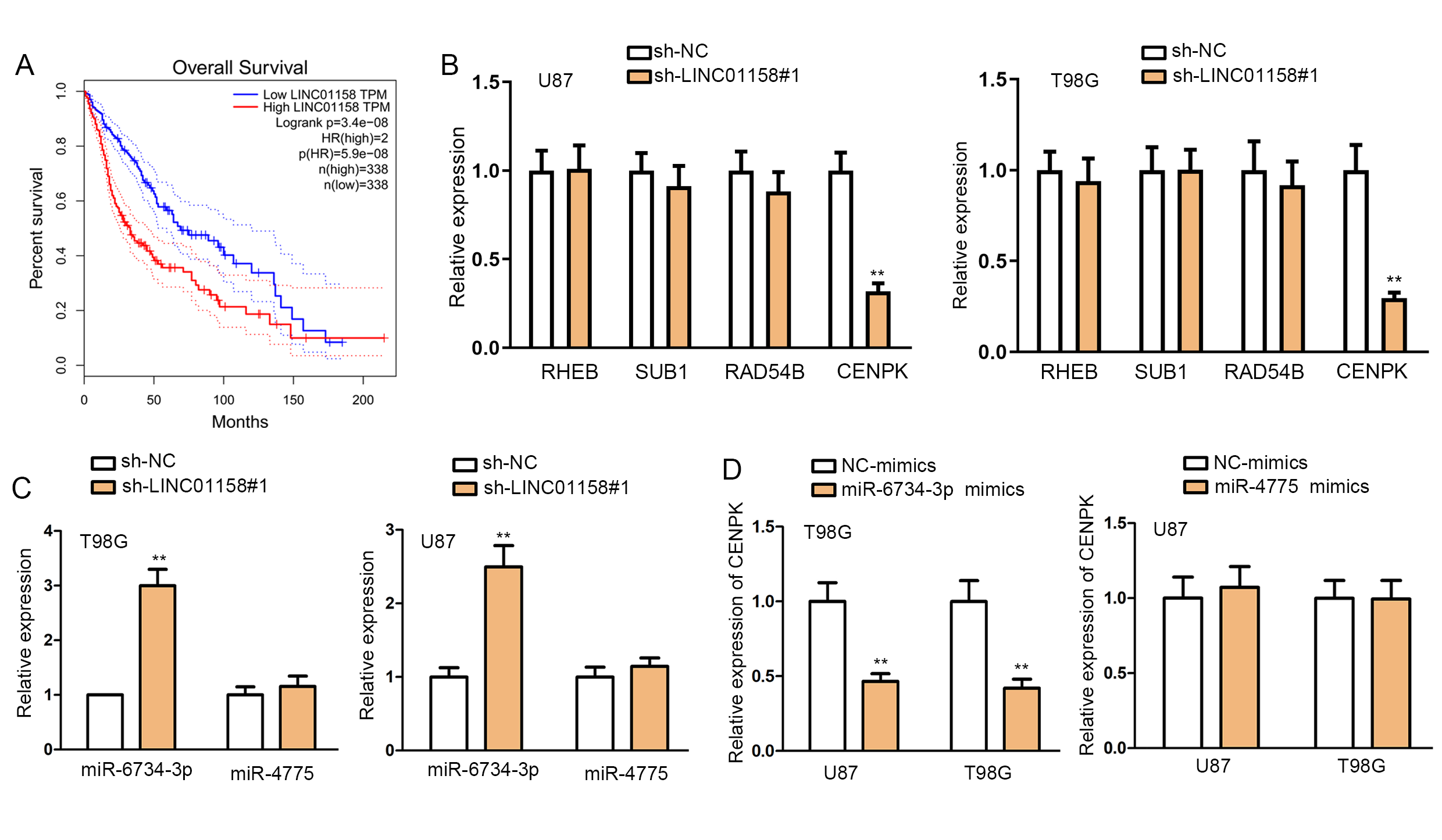

Supplement: Supplementary file 1 — Additional file 1: Figure S1. (A) GEPIA data of the survival curve of patients with glioma (including both GBM and low grade glioma) possessing low or high LINC01158 level. (B) Impact of LINC01158 inhibition on the expression of RHEB, SUB1, RAD54B or CENPK in U87 and T98G cells was assessed by qRT-PCR. (C) The effects of LINC01158 depletion on miR-6734-3p and miR-4775 levels were under qRT-PCR examination. (D) The influence of miR-6734-3p and miR-4775 on the expression of CENPK was under the analysis of qRT-PCR. ** notes P < 0.01. [file 12935_2021_1931_MOESM1_ESM.tif]

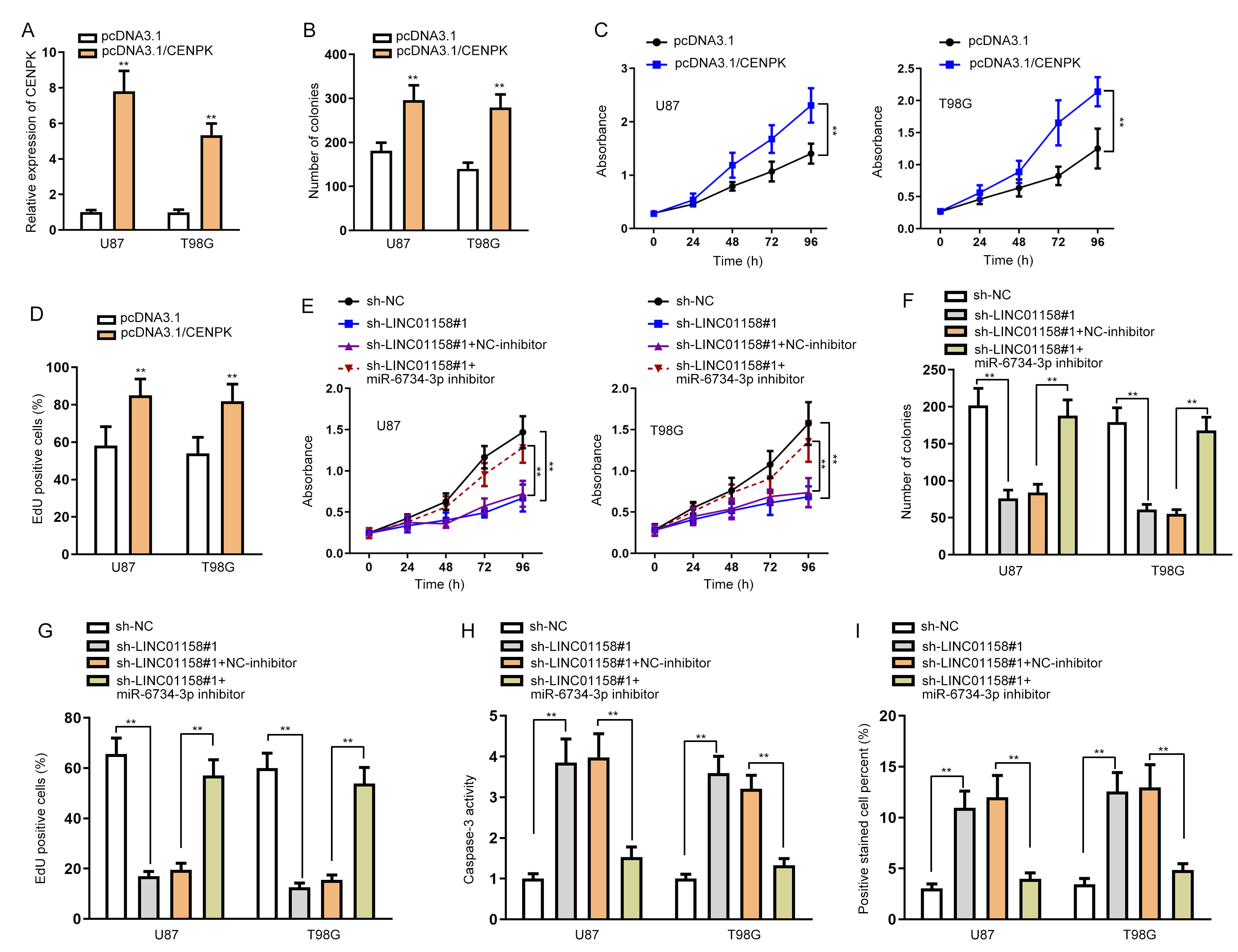

Supplement: Supplementary file 2 — Additional file 2: Figure S2. (A) The expression of CENPK in glioma cells transfected with pcDNA3.1 or pcDNA3.1/CENPK was tested by qRT-PCR. (B-D) The impact of CENPK overexpression on the function of glioma cells was evaluated by colony formation, CCK-8 and EdU assays. (E-I) The rescuing effect of miR-6734-3p inhibition on the function of LINC01158-depleted glioma cells was estimated through CCK-8, colony formation, EdU, caspase-3 activity and TUNEL assays. ** notes P < 0.01. [file 12935_2021_1931_MOESM2_ESM.tif]
